# A-to-I RNA editing in leukemia stem cells - set ADAR1 on the radar

**DOI:** 10.18632/oncotarget.27261

**Published:** 2019-10-22

**Authors:** Qingfei Jiang, Raymond Diep, Catriona Jamieson

**Affiliations:** Division of Regenerative Medicine, Department of Medicine, Moores Cancer Center, University of California, San Diego, La Jolla, California 92093-0820, USA; Division of Regenerative Medicine, Department of Medicine, Moores Cancer Center, University of California, San Diego, La Jolla, California 92093-0820, USA

**Keywords:** cancer stem cell, leukemia stem cell, RNA editing, post-transcriptional regulation

Cancer stem cells (CSCs) or leukemia stem cells (LSCs) in hematological malignancies are a rare cell population characterized by their stem cell-like properties, which lends to their role in disease relapse when treatment is removed [1]. The CSCs are stem cells or early progenitors which undergo oncogenic transformation leading to enhanced self-renewal capacity, quiescence in a protective microenvironment, and resistance to treatment. For the past decades, intense research efforts have focused on deciphering the unique genomic program to maintain CSC “stemness”. This yielded discovery of driver mutations such as *TET2*, *FLT3-ITD*, *JAK2*, and *EZH2* during clonal hematopoiesis. However, until recently, transcriptome dysregulations in CSCs have been often overlooked.

Our laboratory studies the role of double-stranded RNA-specific adenosine deaminase1 (ADAR1) in LSC maintenance in the setting of chronic myeloid leukemia (CML). Conversion of adenosine to inosine (A-to-I) catalyzed by ADAR1 is the most common type of post-transcriptional RNA editing [2]. These transcriptomic alterations (i.e. editome) can affect both coding and non-coding gene expression, as well as microRNA (miRNA) targeting in the rare stem and progenitor cells. In the setting of normal hematopoiesis, A-to-I RNA editing suppresses interferon signaling by distinguishing self and non-self viral RNAs during innate immune response and is crucial for the development of normal hematopoiesis [3–5]. Conversely, malignant ADAR1 activation in cancer cells can be a dominant driver of disease progression and therapeutic resistance [6].

The first evidence that RNA editing plays an important role in LSC function and maintenance was the discovery that the interferon-inducible isoform of ADAR1, ADAR1p150, is highly upregulated in CML LSCs [7]. Additional studies revealed that ADAR1p150 behaves as a self-renewal factor in LSCs through multiple molecular mechanisms, which include inducing *GSK3*β missplicing, altering miRNA biogenesis, and introducing 3′ UTR editing to evade miRNA targeting [1, 8]. We recently reported that ADAR1 has diverse roles in normal HSC and LSC maintenance [8]. We examined the edited miRNome of 1008 miRNAs and found that in normal Hematopoietic stem cell (HSC), ADAR1-mediated RNA editing accelerates cell cycle transit by impairing miR-26a biogenesis and represses *CDKN1A* expression indirectly via *EZH2* methyltransferase. In LSCs, ADAR1 preferentially hyper-edits *MDM2* proto-oncogene at miRNA targeting sites, while correspondingly depletes miR-155 which targets *MDM2.*

The differential target recognition of ADAR1 in HSCs and LSCs could be the result of regulators of ADAR1 activity, or double-stranded RNA structure/substrate abundance and needs to be further investigated. Nonetheless, the overall A-to-I RNA editing levels and targets appear to be cell type and context specific and likely also depend on the cell cycle state. It is particularly challenging to study the human editome due to the variable nature of patient samples. Therefore, sensitive single cell transcriptomic sequencing with sufficient read coverage will be required to reveal the “true” editome within individual LSCs that could be masked by bulk RNA-sequencing.

Similar to DNA mutations, RNA editing likely results in passenger mutations rather than driver mutations. Nonetheless, LSC-specific hyper-RNA editing events can be sensitive biomarkers for LSC detection. For example, *MDM2* is hyper-edited within a small region (approximately 500 nt) and the transcript is more abundant in LSCs compared to normal HSCs, making it an ideal target for standard detection methods in the clinical setting. However, the challenge remains to determine the sensitivity and specificity of RNA hyper-editing to predict outcome and response to treatment in a large cohort of clinical samples.

Dysregulated ADAR1 activity has been observed in 17 cancer types and over 6,000 patient samples, making it an attractive therapeutic target in cancer [6]. More recently, a landmark discovery showed that loss of ADAR1 can sensitize cancer cells to immunotherapy and overcomes the resistance to PD-1 immune checkpoint blockade [9]. Here, ADAR1 depletion results in repolarization of the tumor microenvironment by favoring CD8^+^ cytotoxic T cell infiltration, as well as enhanced dsRNA sensing and the associated inflammation in cancer cells. Thus, targeted therapy blocking ADAR1 activity will provide a general strategy to eliminate LSCs and overcome PD-1 checkpoint blockade. Since ADAR1 activity is also required for HSC self-renewal capacity, any potential inhibitors should also be examined with normal HSCs to ensure they spare the healthy stem cell population.

In conclusion, we discovered LSC-specific hyper-editing of miRNome and 3′UTR regions containing miRNA targeting sites, which leads to enhanced LSC self-renewal. We will focus our future research efforts to better understand the regulatory mechanism and functions of A-to-I RNA editing in the setting of LSCs. Putting ADAR1 on the radar will facilitate improved clinical biomarkers and novel therapeutic targets for cancer research.
